# Synthesis, Photophysical and Electrochemical Properties of a Mixed Bipyridyl-Phenanthrolyl Ligand Ru(II) Heteroleptic Complex Having *trans*-2-Methyl-2-butenoic Acid Functionalities

**DOI:** 10.3390/molecules16108353

**Published:** 2011-09-30

**Authors:** Adewale O. Adeloye

**Keywords:** mixed-ligand Ru(II) complex, extended-π-bond conjugation, spectroscopy, molar extinction coefficient, electrochemistry

## Abstract

In this work, two ligands: 4-(*trans*-2-Methyl-2-butenoic acid)-2,2'-bipyridine) (**L_1_**) and 5-(*trans*-2-methyl-2-butenoic acid)-1,10-phenanthroline (**L_2_**), with the corresponding mixed-ligand heteroleptic Ru(II) complex were synthesized and characterized by FT-IR, ^1^H-, ^13^C-NMR spectroscopy and elemental analysis. The influence of the mixed functionalized polypyridyl ruthenium(II) complex on the photophysical and electrochemical properties were investigated and compared to individual single-ligand homoleptic complexes. Interestingly, the mixed-ligand complex formulated as [RuL_1_L_2_(NCS)_2_] exhibits broad and intense metal-to-ligand charge transfer (MLCT) absorption with a high molar extinction coefficient (λ_max_ = 514 nm, ε = 69,700 M^−1^ cm^−1^), better than those of individual single-ligand complexes, [Ru(L_1_)_2_(NCS)_2_] and [Ru(L_2_)_2_(NCS)_2_], and a strong photoluminescence intensity ratio in the red region at λ_em_ = 686 nm. The electrochemical properties of the complex indicated that the redox processes are ligand-based.

## 1. Introduction

Tremendous interest has been attracted to ruthenium(II) polypyridyl complexes because of their potential applications in molecular electronic devices [1], as DNA structural probes or new therapeutic agents [2,3], and as photosensitizers in the conversion of solar energy to chemical or electrical energy [4]. It has been shown that one of the best way to enhance both the absorption coefficient and red-shift of the metal-to-ligand charge transfer (MLCT) band in a ruthenium-based photosensitizer was to extend the π-conjugation length of the colorant’s ancillary [5] or anchoring [6] ligands. Other classes of ligands such as carboxylated terpyridine and phenanthroline showed enhanced UV-Vis absorption over a broad range due to their large conjugated backbone structure [7]. Over the past decade quite a large amount of data has accumulated on changes in the electrochemical and photophysical properties of complexes effected by substitution in the 2,2'-bipyridine (bpy) or 1,10-phenanthroline (phen) rings, or by replacement of one or both of the pyridine rings with other nitrogen-containing heterocycles [8,9,10,11,12,13]. It has been shown that redox behaviour of these complexes depends on the nature of the ligands, and there is a correlation between their spectroscopic properties and Ru^II^/Ru^III^ redox potential. Further studies of ruthenium(II) complexes are essential to understanding the relationship between structure and function [14].

Based on our earlier studies and recent findings on ruthenium(II) heteroleptic complexes containing mono- and/or oligo- anthracenyl functionalized polypyridine ligands, it was shown that the molar absorption coefficient of ruthenium(II) complexes are not solely dependent on the extended π-conjugation of the ancillary and anchoring ligands, but other factors such as the spatial geometry and molecular aggregation in the molecules also play major roles [15,16,17]. Herein, a further attempt to gain insight into the effects of ligand functionalization and/or mixed-ligand coordination on the photophysical and electrochemical properties of ruthenium(II) polypyridyl complexes is undertaken and discussed.

## 2. Results and Discussion

### 2.1. Chemistry

**Scheme 1:**
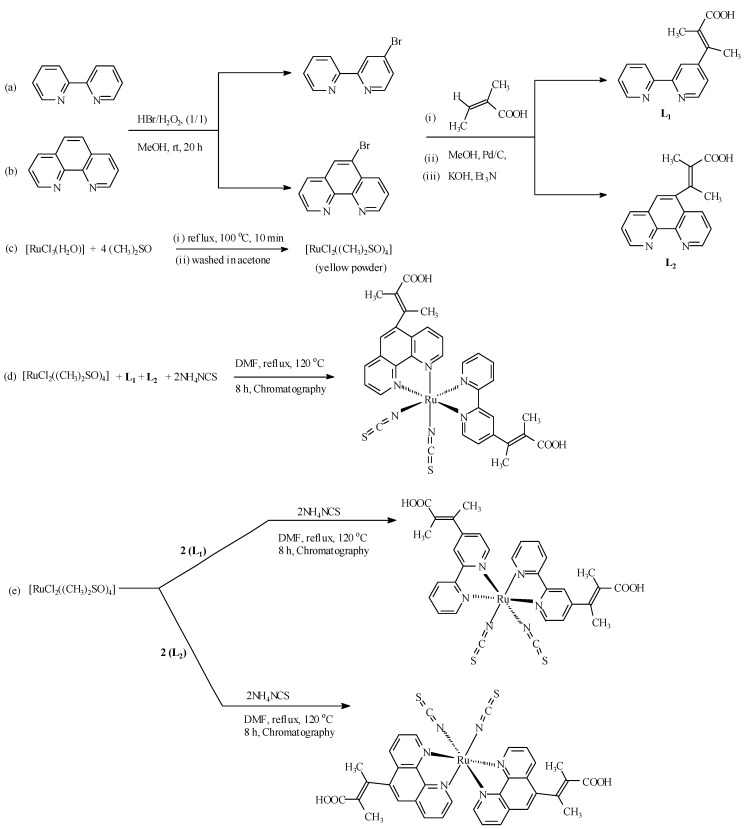
Synthetic pathways for Ligands **L_1_**, **L_2_**, and complexes [RuL_1_L_2_(NCS)_2_], [Ru(L_1_)_2_(NCS)_2_] and [Ru(L_2_)_2_(NCS)_2_].

Scheme 1 (routes a and b) show the two step synthetic pathways for **L_1_** and **L_2_**, and outline the chemistry of the present study. An initial mono-bromination of the starting materials (2,2'-bipyridine and 1,10-phenanthroline) to afford 4-bromo-2,2'-bipyridine and 5-bromo-1,10-phenanthroline was performed using environmentally benign conditions as reported by Vyas and co-workers [18]. A one-step nucleophilic aromatic substitution reaction of the aryl bromide compounds and *trans*-2-methyl-2-butenoic acid in a basic reaction condition using a stochiometric ratio of triethylamine and potassium hydroxide under palladium-carbide catalysis afforded **L_1_** and **L_2_** after extraction into dichloro- methane/chloroform in good yield. The synthesis of the metal precursor [RuCl_2_(dmso)_4_] [19], and the complexes [RuL_1_L_2_(NCS)_2_], [Ru(L_1_)_2_(NCS)_2_] and [Ru(L_2_)_2_(NCS)_2_] followed the general synthetic routes shown in Scheme 1 (routes c–e). Sequential substitution of the DMSO coordinating ligand from the metal precursor with stoichiometric ratio of L_1_, L_2_, and combination of L_1_ and L_2_ led to formation of intermediate complexes [Ru(L_1_)_2_Cl_2_], [Ru(L_2_)_2_Cl_2_] and [RuL_1_L_2_Cl_2_], respectively, which were not isolated. Final reactions were carried out by chloride exchange with thiocyanate groups [20].

### 2.2. Infrared Spectra Studies of the Ligands and Complex

**Figure 1:**
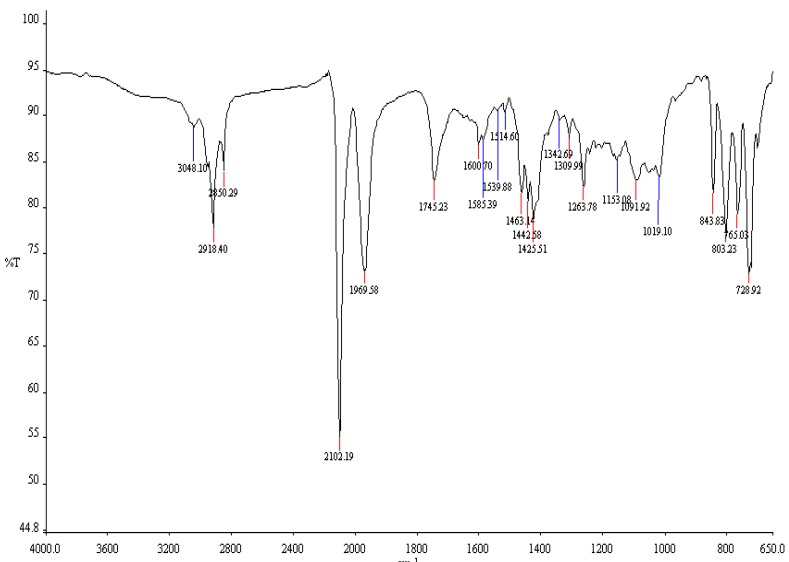
FT-IR spectrum of [RuL_1_L_2_(NCS)_2_] complex.

The infrared absorption spectra of the ligands **L_1_** and **L_2_** and the complex [RuL_1_L_2_(NCS)_2_] share as common characteristic features a series of weak-to-moderate absorptions in the 3,390–3,032 cm^−1^ region, which were assigned to aromatic C–H stretching and/or considered indicative of the presence of O–H_str_ groups in the compounds. The vibrational bands at 2,927, 2,938 cm^−1^ in **L_1_** and **L_2_**, respectively, show the presence of the aliphatic C–H_str_ bonds of the methyl, methylene and/or the methine groups in the compounds [21]. A slight difference shift to lower frequency (~10 cm^−1^) may be due to spatial arrangement and/or substitution pattern of the alkyl groups in the [RuL_1_L_2_(NCS)_2_] complex. The strong intense band at 2,102 cm^−1^ present in the complex (Figure 1), was assigned to the stretch vibrational modes due to N-coordinated υ(CN) of the thiocyanate group. This band is conspicuously absent in the spectra of the ligands.

The spectra of the two ligands and the complex display asymmetric carboxylate stretching bands ν_asym_(COO^−^) at 1,965 cm^−1^ for **L_1_**, and 1,973 cm^−1^ for **L_2_**, while the complex showed a very similar but, more intense vibrational frequency band at 1,969 cm^−1^. It is interesting to note that upon addition of a 1 mL volume of 20% conc. sulphuric acid to 20 mg of [RuL_1_L_2_(NCS)_2_] complex, a strong band at 1,745 cm^−1^ was observed; this is indicative of the protonation and/or deprotonation of the carboxylate group on either the bipyridine and phenanthroline ligands. If the ligands were partially deprotonated, ν_asym_(COO^−^) would be expected at ~1,600 cm^−1^ [22]. However, C=C and C=N stretching bands of the bipyridyl and phenanthrolyl ligands also appear near 1,600 cm^−1^ making it difficult to determine if the H-bpy and/or H-phen ligand is fully or only partially protonated on this basis. Aside from the small variations in the ν_asym_(COO^−^) bands, the IR spectra of the **L_1_**, **L_2_** ligands and [RuL_1_L_2_(NCS)_2_] complex are in very good agreement. In the fingerprint region, comparison of the infrared spectra of **L_1_**, **L_2_** with that of the [RuL_1_L_2_(NCS)_2_] complex shows vibrational frequency bands at 757, 619 cm^−1^ for **L_1_**; 843, 734 cm^−1^ for **L_2_**, and four bands of almost equal intensity at 843, 803, 765 and 728 cm^−1^ for the [RuL_1_L_2_(NCS)_2_] complex. These bands are associated with the mono-substitution pattern of the anchoring *trans*-2-methyl-2-butenoic acid on the bipyridyl and phenanthrolyl ligands, and also confirmed the mixed-ligand coordination of the [RuL_1_L_2_(NCS)_2_] complex. 

### 2.3. ^1^H and ^13^C-NMR Spectroscopic Studies

**Figure 2:**
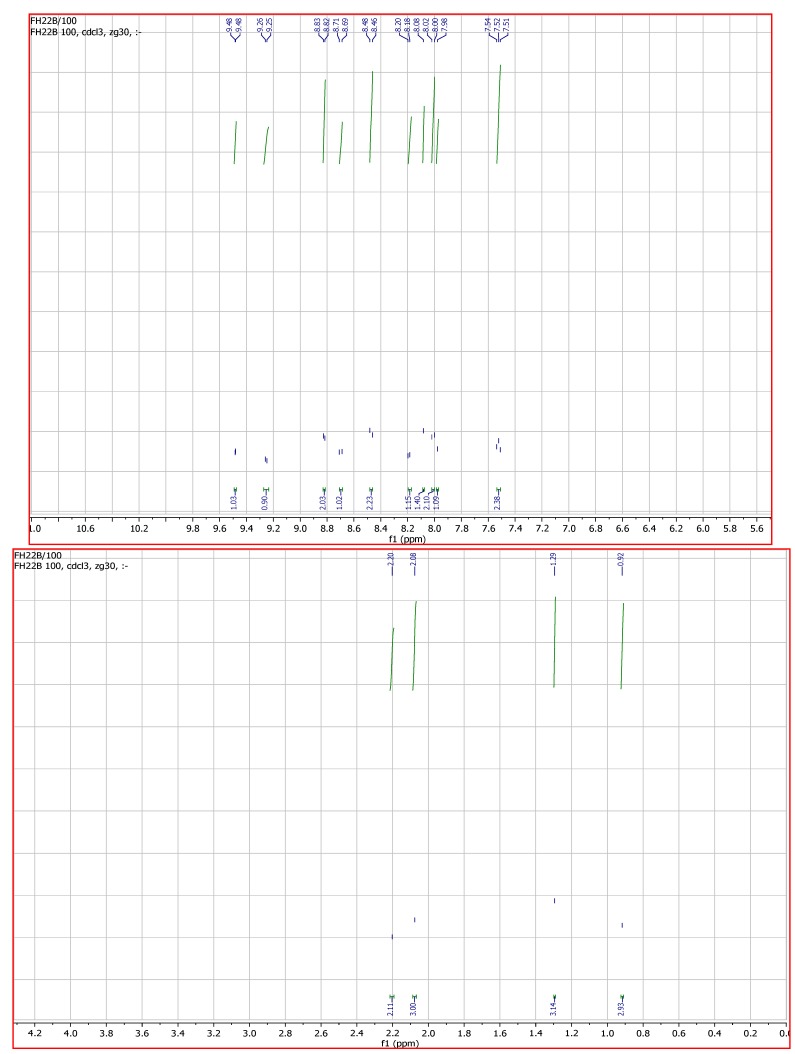
^1^H-NMR spectrum of [RuL_1_L_2_(NCS)_2_] in CDCl_3_, (δ) ppm.

The aromatic region of the ^1^H-NMR spectrum of **L_1_** gave six peaks at δ 8.66 (d, 1H), 8.41 (d, 1H), 7.86 (dd, 1H), 7.36 (dd, 1H), 1.73 (s, CH_3_) and 1.66 (d, CH_3_) ppm. The ^1^H peaks are very similar to those of the bromobipyridine starting material. The main difference is due to the appearance of the methyl resonance in the aliphatic region of the spectrum. The ^13^C-NMR spectrum gave the anticipated peaks at 169.76, 156.21, 149.98, 137.86, 136.88, 129.74, 124.81, 121.32, 14.81, and 12.71. The bipyridine peaks, due to chemical equivalency, were observed in the 156–128 ppm range. The peak at 169.76 ppm was assigned to the carbonyl carbon; the two peaks at 124.81 and 121.32 were assigned to the alkenyl carbons, while the *trans*-methyl group peaks were found at 14.81 and 12.71 ppm. The ^1^H-NMR of **L_2_** shows six signals in the aromatic region at δ 11.32 (br, OH), 8.98 (t, 2H), 7.92 (t, 2H), 7.45 (s, 1H), and 7.35 (2d, 2H), that were assigned to the phenanthroline protons with indication of the presence of a water molecule. The two signals in the aliphatic region at δ 1.68 (s, CH_3_) and 1.59 (d, CH_3_) were assigned to the *trans*-methyl protons of the carboxylic acid group. The ^13^C spectrum of **L_2_** was similar to that of **L_1_**, except for the downfield deshielding effect on the carbonyl carbon to 172.83 ppm. Other signals in the spectrum correspond to that of a phenanthroline and *trans*-2-methyl-2-butenoic acid moieties [16,17]. The proton NMR spectrum of the [RuL_1_L_2_(NCS)_2_] complex in CDCl_3_ (Figure 2) is consistent with the structure shown in Scheme 1.

The presence of two different ancillary coordination ligands, such that all the protons are found in electronically different environments, complicated both the aromatic and the aliphatic regions of the spectrum, which showed ten distinct proton signals between δ 9.48–7.53 ppm integrating for fourteen protons. By comparison with the **L_2_** ligand, the phenanthroline protons were observed as doublets at δ 9.25 and 9.48 ppm for H-2 and H-9 respectively. Two multiplet peaks at δ 8.01 and 7.53 ppm, integrating for four protons, were assigned to H-3,8; and H-4,7 respectively, while the broad signal at δ 8.08 ppm, integrating for one proton, was assigned to H-6. The unsubstituted arm of the **L_1_** ligand showed a doublet signal at 8.47 ppm, assigned to the H-3', H-4' protons, while the H-5' and H-6' proton signals were found at 8.82 ppm. A doublet peak at δ 8.70 ppm was assigned to H-6, and H-5, H-3 were assigned to a triplet at δ 8.19 ppm and a singlet at 7.98 ppm respectively. In the aliphatic region, four broad singlet peaks at δ 2.20, 2.08, 1.29 and 0.92 ppm were unambiguously assigned to the *trans-*methyl groups in the complex. In comparison to the individual ligands, the proton peaks in the complex were shifted downfield. The deshielding pattern may be ascribed to the lone pair-lone pair electron donation of the nitrogen to the *d*-orbital of the ruthenium metal. The ^13^C-NMR spectrum data of the complex could not be adequately assigned to individual carbon atoms due to its complexity and poor signal resolutions.

### 2.4. Electronic Absorption and Emission Spectra

#### 2.4.1. Electronic Absorption Spectroscopy

**Figure 3:**
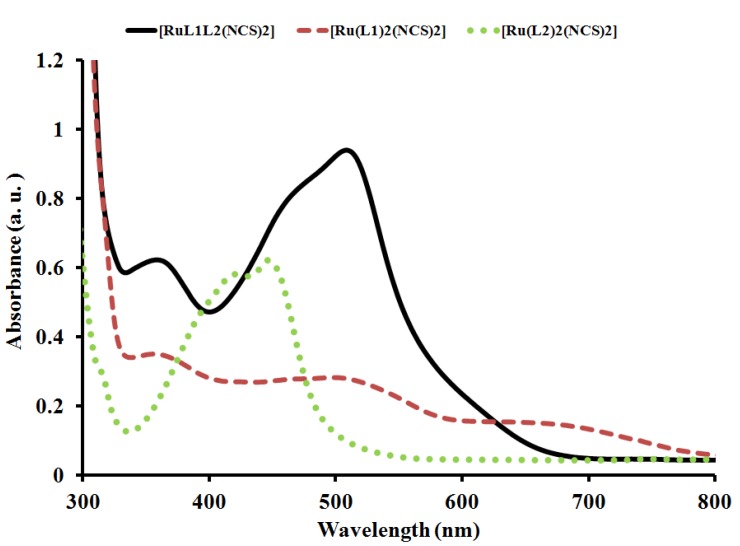
Comparison of the UV-Vis absorption spectrum of [RuL_1_L_2_(NCS)_2_] (solid line) with [Ru(L_1_)_2_(NCS)_2_] (dash line) and [Ru(L_2_)_2_(NCS)_2_] (dot line) at a concentration of 1 × 10^−3^ M^−1^ in DMF solution showing effect of extended π-conjugation and mixed-ligand coordination.

**Figure 4:**
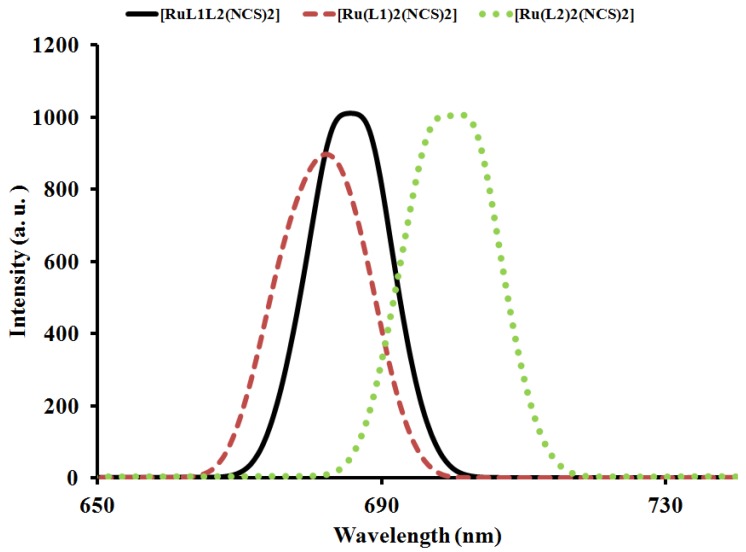
Comparison of emission spectral of [RuL_1_L_2_(NCS)_2_] with [Ru(L_1_)_2_(NCS)_2_] and [Ru(L_2_)_2_(NCS)_2_] in DMF solution.

The UV-Vis absorbance and emission spectra of complex [RuL_1_L_2_(NCS)_2_] were recorded at room temperature in aerated DMF solution and are shown in Figure 3 and Figure 4. The complex exhibits a very broad and intense metal-to-ligand charge (MLCT) absorption band throughout the visible region of the spectrum (400–700 nm), characteristic of many other ruthenium(II) polypyridyl complexes, and which can be assigned to electronic transitions from the Ru^II^ based t_2_g orbital to the ligand based π* orbitals. The molar extinction coefficient of complex [RuL_1_L_2_(NCS)_2_] at its maximum (514 nm) is 6.97 × 10^4^ M^−1^ cm^−1^, precisely 48,950 and 21,552 M^−1^ cm^−1^ larger than that of [Ru(L_1_)_2_(NCS)_2_] (2.04 × 10^4^ M^−1^ cm^−1^) at 508 nm and [Ru(L_2_)_2_(NCS)_2_] (4.82 × 10^4^ M^−1^ cm^−1^) at 446 nm, respectively. The mixed-ligand nature of complex [RuL_1_L_2_(NCS)_2_] unlike [Ru(L_2_)_2_(NCS)_2_], produces multiple MLCT transitions in the visible, *i.e.*, Ru→**L_1_** and Ru→**L_2_** with broad absorption at 352 nm (4.81 × 10^4^ M^−1^ cm^−1^), thus enhancing the oscillator strength in the blue and green portions of the spectrum relative to [Ru(L_1_)_2_(NCS)_2_]. These features have been found as a means of improving light absorption cross-sections at higher energy in similar complexes [23]. The intense absorption band in the UV region around 280–300 nm (not shown) were assigned to the intraligand π→π* transitions of **L_1_** and **L_2_** ligands. The lower-energy absorption in complex [RuL_1_L_2_(NCS)_2_], was enhanced due to the presence of the electron withdrawing nature of the carboxylic groups, which lowers the energy of the π* orbital of the bipyridine and phenanthroline ligands, and a further stability of the complex provided by the *trans* nature of the methyl groups.

#### 2.4.2. Emission Study

The emission spectrum of complex [RuL_1_L_2_(NCS)_2_] compared with those of complexes [Ru(L_1_)_2_(NCS)_2_] and [Ru(L_2_)_2_(NCS)_2_] is displayed in Figure 4. Upon excitation into the ^1^LC and ^1^MLCT bands, (λ_exc._ = 500 nm), the complex displays appreciable luminescence at room temperature. An emission wavelength maximum was found at 686 nm which is blue-shifted and of lower intensity at 682 nm for complex [Ru(L_1_)_2_(NCS)_2_], whereas a red-shift with enhanced intensity at 700 nm was observed for complex [Ru(L_2_)_2_(NCS)_2_]. It is well known that conjugated functional organic molecules are useful for the study of electron transport at the molecular scale and that the use of fused-ring systems is a powerful and practical approach [24,25].

The luminescent properties of a complex as well as its ability to play the role of excited state reactant or product are related to the energy ordering of its low energy excited state and, particularly, to the orbital nature of its lowest excited state. It could be seen in the complex that the choice of ligands, has significant effects, which in turn influence the energy positions of the metal centre (MC) and ligand centre (LC), as well as the metal-to-ligand transfer (MLCT). The energy of the MC excited state depends on the ligand field strength, which in turn depends on the σ-donor and π-acceptor properties of the ligands, the steric crowding around the metal (that can preclude a sufficiently close approach between metal and ligand) [26,27,28], and the bite angle of the polydentate ligands (which in some cases cannot be optimized because of molecular constraint) [29]. The energy of the LC excited state depends on the intrinsic properties of the ligands, such as the HOMO-LUMO energy gap and the singlet-triplet splitting. It has been shown that the energy of the MLCT excited state depends on the reduction potential of the ligand involved in the MLCT transition, the oxidation potential of the metal in the complex (which is affected by the electron donor and acceptor properties of all the ligands), and by the charge separation caused by the transition. In the complex [RuL_1_L_2_(NCS)_2_], the intense emission is a significant contribution to the excited state from an interaction between the metal *d*-orbital and the ligand π-systems [30].

### 2.5. Electrochemical Study

**Figure 5:**
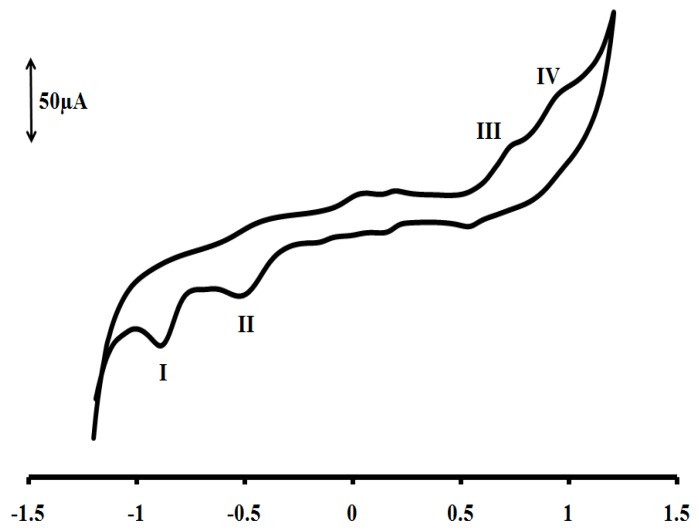
Cyclic voltammogram for [RuL_1_L_2_(NCS)_2_] complex at 1 × 10^−3^ M in freshly distilled DMF containing 0.1 M TBABF_4_ supporting electrolyte. Step potential = 5 mV, amplitude = 50 mV *vs*. Ag|AgCl, frequency = 10 Hz. Scan rate = 100 m Vs^−1^*vs*. Ag|AgCl.

**Figure 6:**
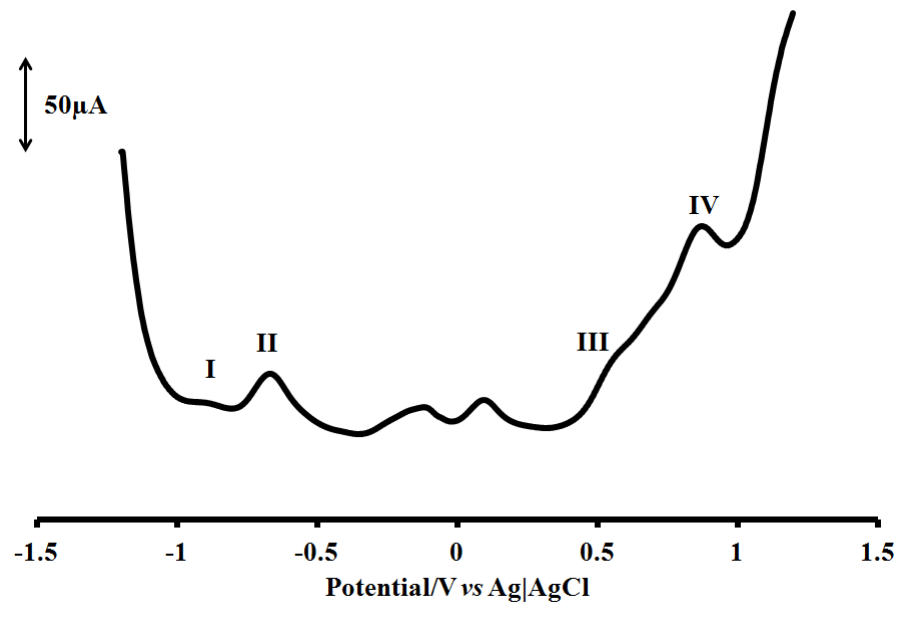
Square wave voltammogram for [RuL_1_L_2_(NCS)_2_] complex at 1 × 10^−3^ M in freshly distilled DMF containing 0.1 M TBABF_4_ supporting electrolyte. Step potential = 5 mV, amplitude = 50 mV *vs*. Ag|AgCl, frequency = 10 Hz. Scan rate = 100 m Vs^−1^*vs*. Ag|AgCl.

The cyclic and square wave voltammograms of the complex [RuL_1_L_2_(NCS)_2_] were examined in the potential range +1.5 to −1.5 V and at a scan rate 50 mV s^−1^ using Ag|AgCl electrode in DMF solvent with 0.1 M tetrabutylammonium hexafluorophosphate as supporting electrolyte (Figure 5 and Figure 6).

The voltammograms display the Ru(III)/Ru(II) couple at positive potential and ligand based reduction couples at negative potentials. The quasi-reversible one-electron oxidation process **IV** at E_1/2_ = +0.85 V, was assigned to the metal centre Ru(III)/Ru(II) wave couple [31]. Some unidentified oxidation peak processes were observed, but were most pronounced in process **III** (E_1/2_ = +0.52 V) which were tentatively ascribed to the ring oxidation of the bipyridine and/or phenanthroline ligands and, as well as the carboxylate ions present in the complex. Two irreversible waves were observed at −0.92 and −0.70 V for processes **I** and **II** respectively. The more positive reduction potential may be assigned to the contribution from **L_2_** due to an additional (C=C) conjugative π-bond as found in the structure of phenanthroline since both ligands contain the electron withdrawing COOH group being responsible for lowering the LUMO levels.

## 3. Experimental

### 3.1. Materials and General Physical Measurements

All chemical and reagents were analytically pure and used without further purification. 4-Bromo-2,2'-bipyridine and 5-bromo-1,10-phenanthroline were synthesized as described in the literature [18]. 4-(*trans*-2-Methyl-2-butenoic acid)-2,2'-bipyridine (**L_1_**) and 5-(*trans*-2-methyl-2-butenoic acid)-1,10-phenanthroline (**L_2_**) were synthesized following the literature procedure [15] with slight modifications (Scheme 1). All thin layer chromatography (TLC) analyses were done on aluminium sheets precoated with normal phase silica gel 60 F_254_ (Merck, 0.20 mm thickness), unless otherwise stated. The TLC plates were developed using any of the following solvent systems: Solvent system A: dichloromethane-methanol (9:1); Solvent system B: dichloromethane-methanol (7:3); Solvent system C: diethyl ether-methanol (1:1). Gel filtration was performed using Sephadex LH-20 previously swollen in specified solvent (s) prior to loading of extract onto the column (3.5 cm × 8.5 cm).

Melting points were determined using a Gallenkamp electrothermal melting point apparatus. Microanalyses (C, H, N, and S) were carried out with a Fisons elemental analyzer and infrared spectra were obtained with KBr discs on a Perkin Elmer System 2000 FT-IR spectrophotometer. UV-Vis and fluorescence spectra were recorded in a 1 cm path length quartz cell on a Perkin Elmer Lambda 35 spectrophotometer and Perkin Elmer Lambda 45 spectrofluorimeter, respectively. ^1^H- and ^13^C-NMR spectra were run on a Bruker EMX spectrometer operating at 400 MHz for ^1^H and 100 MHz for ^13^C. The chemical shift values were reported in parts per million (ppm) relative to (TMS) as internal standard. Chemical shifts were reported for the ligands and complex with respect to CDCl_3_ at δc 77.00 and δ_H_ CDCl_3_ at 7.26. Electrochemical experiment was performed using a PGSTAT 302 Autolab potentiostat (EcoChemie, Utrecht, The Netherlands) driven by the general purpose Electrochemical System data processing software (GPES, software version 4.9). A conventional three-electrode system was used. The working electrode was a bare glassy carbon electrode (GCE), Ag|AgCl wire and platinum wire were used as the pseudo reference and auxiliary electrodes, respectively. The potential response of the Ag|AgCl pseudo-reference electrode was less than the Ag|AgCl (3 M KCl) by 0.015 ± 0.003 V. Prior to use, the electrode surface was polished with alumina on a Buehler felt pad and rinsed with excess millipore water. All electrochemical experiments were performed in freshly distilled dry DMF containing TBABF_4_ as supporting electrolyte.

### 3.2. Synthesis of 4-(trans-2-Methyl-2-butenoic acid)-2,2'-bipyridine *(**L_1_**)*

4-Bromo-2,2'-bpy (1.05 g, 3.38 mmol) and *trans*-2-methyl-2-butenoic acid (0.34 g, 3.38 mmol) were dissolved in MeOH (40 mL) in a 250 mL flask. Et_3_N (1.0 mL and palladium-carbide (0.050 g) were added and the mixture was reflux for 8 h at a temperature between 110−120 °C. The reaction was allowed to cool to room temperature, and filtered, and the solvent removed under reduced pressure. The residue was dissolved in degassed water and then extracted with chloroform. The chloroform extract was concentrated *in vacuo* to give a brilliant colourless liquid which solidified after about 48 h at room temperature. The resultant residue was recrystallized from Et_2_O (30 mL). White crystalline solid; yield: 0.90 g (67%); melting point: ND; IR (KBr, ν_cm_^−1^): 3,054, 2,927, 2,676, 1,965, 1,690, 1,648, 1,581, 1,559, 1,456, 1,419, 1,346, 1,251, 1,141, 1,089, 1,040, 992, 893, 757, 653, 631, 619, 555. ^1^H-NMR (DMSO): δ 8.66 (d, *J* = 4.0 Hz, H-6, 6'), 8.41 (d, *J* = 8.0 Hz, H-3, 3'), 7.86 (dd, *J* = 7.6, 8.0 Hz, H-5, 5'), 7.36 (dd, *J* = 5.2, 7.2 Hz, H-4'), 1.73 (s, CH_3_), 1.66 (d, CH_3_). ^13^C-NMR (DMSO): δ 169.76, 156.21, 149.98, 137.86, 136.88, 129.74, 124.81, 121.32, 14.81, 12.71. Elemental Analysis: Calculated for C_15_H_14_N_2_O_2_: H 5.55, C 70.85, N 11.02; required H 5.55, C 70.60, N 11.43. 

### 3.3. Synthesis of 5-(trans-2-Methyl-2-butenoic acid)-1,10-phenanthroline *(**L_2_**)*

5-Bromo-1,10-phenanthroline (1.0 g, 3.86 mmol) and *trans*-2-methyl-2-butenoic acid (0.39 g, 3.86 mmol) were dissolved in MeOH (40 mL) in a 250 mL flask. Et_3_N (1.0 mL) and palladium-carbide (0.050 g) were added and the mixture was refluxed for 8 h at a temperature between 110−120 °C. The reaction workup was as reported for L_1_ above. White-pink crystalline solid; yield: 0.74 g (53%); melting point: ND; IR (KBr, ν_cm_^−1^): 3,419, 3,032, 2,929, 1,694, 1,652, 1,619, 1,589, 1,561, 1,506, 1,420, 1,385, 1,343, 1,256, 1,219, 1,140, 1,093, 1,080, 1,037, 1,015, 843, 766, 734, 769, 625, 530. ^1^H-NMR (CDCl_3_): δ 11.33 (br, OH), 8.98 (t, *J* = 4.0 Hz, 2H, H-2, 9), 7.92 (d, *J* = 8.0 Hz, 2H, H-4, 7), 7.45 (s, 1H, H-6), 7.35 (2d, *J* = 4.4 Hz, 2H, H-3,8), 1.68 (s, 3H, CH_3_), 1.59 (d, *J* = 6.8 Hz, 3H, CH_3_). ^13^C-NMR (CDCl_3_): δ 172.83, 150.39, 150.30, 146.30, 146.21, 138.79, 136.13, 128.81, 128.72, 126.68, 126.59, 123.27, 123.19, 14.74, 12.14. Elemental Analysis: Calculated for C_17_H_14_N_2_O_2_: H 5.07, C 73.37, N 10.07; required H 4.98, C 73.64, N 10.23.

### 3.4. Synthesis of cis-Dithiocyanato-4-(trans-2-methyl-2-butenoic acid)-2,2'-bipyridyl-5-(trans-2-methyl-2-butenoic acid)-1,10-phenanthrolyl-ruthenium(II) complex *[**RuL_1_L_2_(NCS)_2_**]*

In a 250 mL flask, [RuCl_2_(dmso)_4_] (0.44 g, 0.92 mmol) was dissolved in *N,N*-dimethylformamide (40 mL) followed by the addition of ligands **L**_1_ (0.23 g, 0.92 mmol) and **L**_2_ (0.26 g, 0.92 mmol). The mixture was refluxed initially at 120 °C for 2 h in the dark and an excess of NH_4_NCS (0.70 g, 10.40 mol) was then added. The reaction was left to reflux for an additional 10 h. The solution was allowed to cool to room temperature and filtered to remove unreacted starting material. The filtrate was concentrated to dryness and 0.05 M NaOH solution (40 mL) was added to give a dirty brown precipitate which was filtered off. The pH of the resulting solution was adjusted to 3 with 0.5 M HNO_3_. The solution was left to stand in the fridge (−2 °C) for 12 h before being filtered and concentrated *in vacuo*. The crude residue was adsorbed onto Sephadex LH-20 adsorbent in a glass column and eluted using solvent system C (250 mL). Dark brown solid; melting point: >250 °C; yield: 0.37 g (22%); IR (KBr) ν_max_/cm^−1^: 3,048, 2,918, 2,850, 2,102, 1,969, 1,745, 1,600, 1,585, 1,539, 1,514, 1,463, 1,442, 1,425, 1,342, 1,309, 1,263, 1,153, 1,091, 1,019, 843, 803, 765, 728. UV-Vis (λ_max_/nm, ε = ×10^4^ M^−1^ cm^−1^, DMF): 352 (3.55), 514 (6.97). Emission wavelength: (λ_exc._ = 500 nm, λ_em_ = 686 nm). ^1^H-NMR (CDCl_3_): δ 9.48 (d, *J* = 4 Hz, 1H, H-9), 9.25 (d, *J* = 4 Hz, 1H, H-2), 8.82 (d, *J* = 4 Hz, 2H, H-5',6'_bpy_), 8.70 (d, *J* = 8.0 Hz, 1H, H-6_bpy_), 8.47 (d, *J* = 8.0 Hz, 2H, H-3',4'_bpy_), 8.19 (d, *J* = 8.0 Hz, 1H, H-5_bpy_), 8.08 (s, 1H, H-6_phen_), 8.01 (d, 2H, H-3,8_phen_), 7.98 (s, 1H, H-3_bpy_), 7.53 (m, 2H, H-4,7_phen_), 2.20 (s, 2H), 2.08 (s, 3H), 1.29 (s, 3H), 0.92 (s, 3H). ^13^C-NMR (CDCl_3_): ND. Cyclic voltammetry Data: Ru^2+^/Ru^3+^ = +0.85 V; E_anodic_ = +0.53; E_cathodic_ = −0.70, −0.92 V. Elemental Analysis: Calculated for RuC_34_H_28_N_6_O_4_S_2_: H 3.76, C 54.46, N 11.21, S 8.55; required H 3.87, C 54.79, N 11.56, S 8.44. 

### 3.5. Synthesis of cis-Dithiocyanato-bis-4-(trans-2-methyl-2-butenoic acid)-2,2'-bipyridyl)-ruthenium(II) complex *[**Ru(L_1_)_2_(NCS)_2_**]*

In a 250 mL flask, [RuCl_2_(dmso)_4_] (0.25 g, 0.51 mmol) was dissolved in *N,N*-dimethylformamide (40 mL) followed by the addition of ligand **L_1_** (0.30 g, 1.02 mmol). The mixture was refluxed initially at 120 °C for 2 h in the dark and then an excess of NH_4_NCS (0.70 g, 10.40 mol) was added and the same procedure as described above was followed. Dark brown solid; melting point: >250 °C, yield: 0.07 g (51%); IR (KBr) ν_max_/cm^−1^: 3,550, 3,479, 3,237, 2,923, 2,851, 2,106, 1,638, 1,617, 1,460, 1,443, 1,420, 1,309, 1,265, 1,243, 1,155, 1,084, 1,022, 800, 760, 728, 620, 474, 421. UV-Vis (λ_max_/nm, ε = ×10^4^ M^−1^ cm^−1^, DMF): 346 (2.52), 497 (2.06), 665 (1.09), 740 (0.73). Emission wavelength: (λ_exc._ = 500 nm, λ_em_ = 682 nm). ^1^H-NMR (CDCl_3_): δ 8.93 (br, d, 1H), 8.89 (br, s, 2H), 8.50 (d, 2H), 8.11 (dt, 3H), 8.12 (s, 1H), 8.04 (d, 1H), 7.97 (d, 1H), 7.66 (br, s, 2H), 7.53 (br, s, 1H), 2.17 (br, s, 3H), and 1.25 (s, 9H). ^13^C-NMR (CDCl_3_): ND. Elemental Analysis: Calculated for RuC_32_H_28_N_6_O_4_S_2_: H 3.89, C 52.96, N 11.58, S 8.83; required H 4.12., C 52.57, N 11.55, S 8.69. 

### 3.6. Synthesis of cis-Dithiocyanato-bis-5-(trans-2-methyl-2-butenoic acid)-1,10-phenanthrolyl-ruthenium(II) complex *[**Ru(L_2_)_2_(NCS)_2_**]*

In a 250 mL flask, [RuCl_2_(dmso)_4_] (0.44 g, 0.90 mmol) was dissolved in *N,N*-dimethylformamide (40 mL) followed by the addition of ligand **L_2_** (0.50 g, 1.79 mmol). The mixture was refluxed initially at 120 °C for 2 h in the dark and then an excess of NH_4_NCS (0.27 g, 3.59 mol) was added and the same procedure as described above was followed. Orange solid; melting point: 201–204 °C; yield: 0.38 g (38%); IR (KBr) ν_max_/cm^−1^: 3,423, 3,058, 2,926, 2,856, 2,086, 2,058, 1,978, 1,631, 1,427, 1,384, 1,221, 1,205, 1,147, 1,056, 879, 844, 773, 721, 621, 559. UV-Vis (λ_max_/nm, ε = ×10^4^ M^−1^ cm^−1^, DMF): 416 (4.49), 446 (4.82). Emission wavelength: (λ_exc._ = 500 nm, λ_em_ = 700 nm). ^1^H-NMR (CDCl_3_): δ 8.77 (d, *J* = 8.0 Hz, 4H), 8.38 (s, 2H), 8.08 (d, *J* = 4.8 Hz, 4H), 7.76 (dd, *J* = 5.2, 8.0 Hz, 4H), 3.40 (s, 6H). Selected aromatic ^13^C-NMR data (CDCl_3_): 153.62, 148.13, 137.69, 131.33, 128.92 and 127.17 Elemental Analysis: Calculated for RuC_36_H_28_N_6_O_4_S_2_: H 3.65, C 55.88, N 10.86, S 8.29; required H 3.32, C 55.95, N 10.51, S 8.59.

## 4. Conclusions

In conclusion, an easy to synthesize mixed-ligand heteroleptic ruthenium(II) complex [RuL_1_L_2_(NCS)_2_] with broad spectral bandwidth and visible light absorption properties was designed, synthesized and its photophysical and electrochemical properties investigated. It was shown that a synergy effect brought about by the individual ligands has great influence on the photophysical and the electrochemical properties of the complex, which are better than those of the individual single-ligand homoleptic complexes: [Ru(L_1_)_2_(NCS)_2_] and [Ru(L_2_)_2_(NCS)_2_]. Particularly for this molecule, further work to establish the solar-to-electrical energy conversion efficiency (σ) in its dye-sensitized solar cells (DSSCs) is ongoing in our laboratory. However, in terms other characteristics of ruthenium(II) polypyridine complexes, the high redox potentials, broad visible absorption and high luminescence intensity render the new complex has great potential for use in biomedical applications, involving electrochemical or photochemical detection methods, and/or therapeutic applications. In this context, the carboxylic acid group allows for further functionalization as required to attach the complexes to various substrates and biomolecules.

## Supplementary Materials

Supplementary File 1
