# Supplementary material for: Synthesis, Photophysical and Electrochemical Properties of a Mixed Bipyridyl-Phenanthrolyl Ligand Ru(II) Heteroleptic Complex Having trans-2-Methyl-2-butenoic Acid Functionalities
Source: Molecules. 2011 Sep 30;16(10):8353–67. doi: 10.3390/molecules16108353 (PMC6264457; doi:10.3390/molecules16108353)

Supplementary Information

Synthesis, Photophysical and Electrochemical Properties of a Mixed Bipyridyl-Phenanthrolyl Ligands Ru(II) Heteroleptic Complex Having 2-Methy-2-butenoic Acid Functionality

Adewale O. Adeloye

Department of Chemistry, Faculty of Science and Agriculture, University of Fort Hare, P.M.B. X1314, Alice 5700, South Africa; E-Mail: aadeloye@ufh.ac.za; Tel.: +27-761-423-313; Fax: +27-406-022-366.

S1. ^{1}H-NMR spectrum of **L_1**.

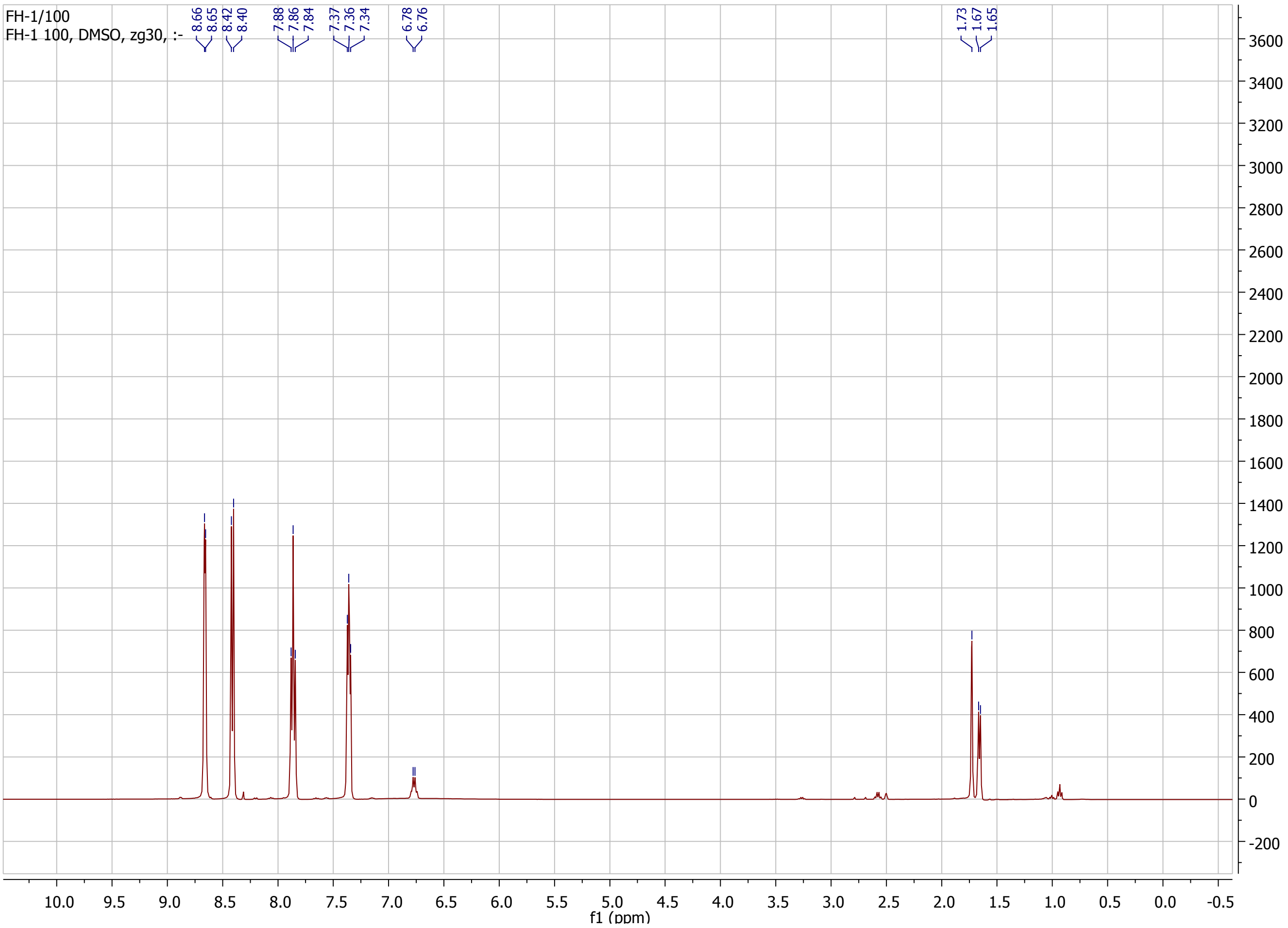

S1. *Cont.*

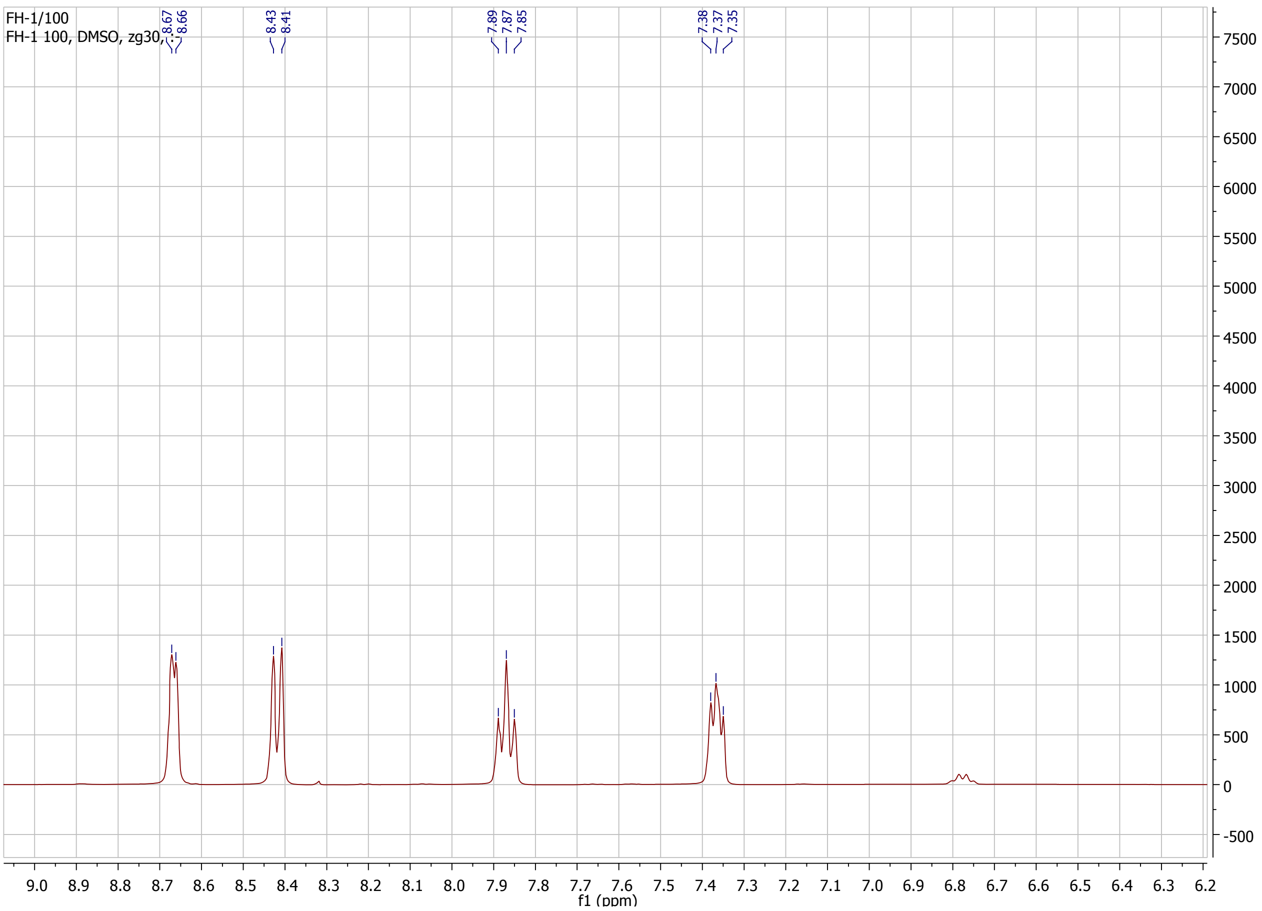

S2. ^{13}C-NMR spectrum of $\mathbf{L_1}$.

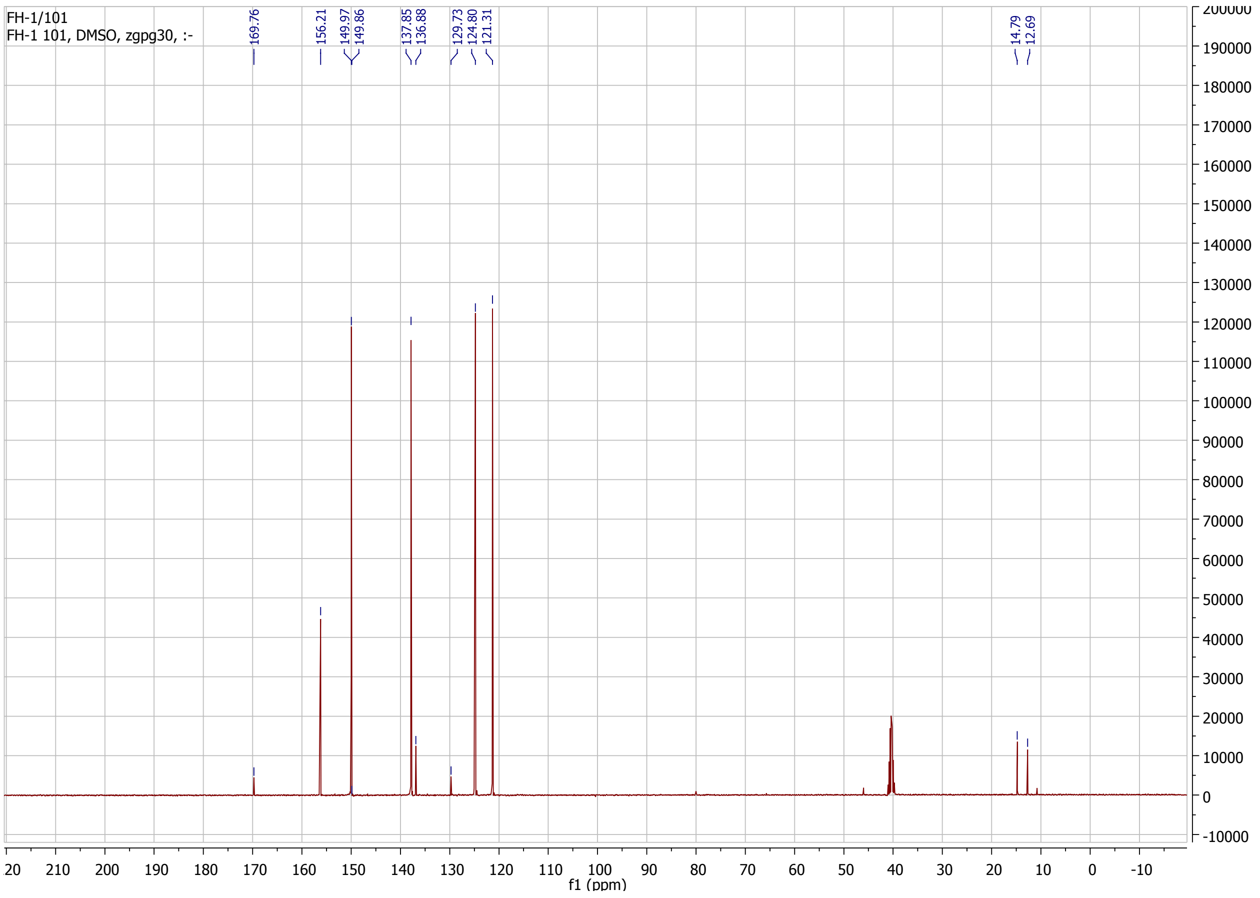

S2. *Cont.*

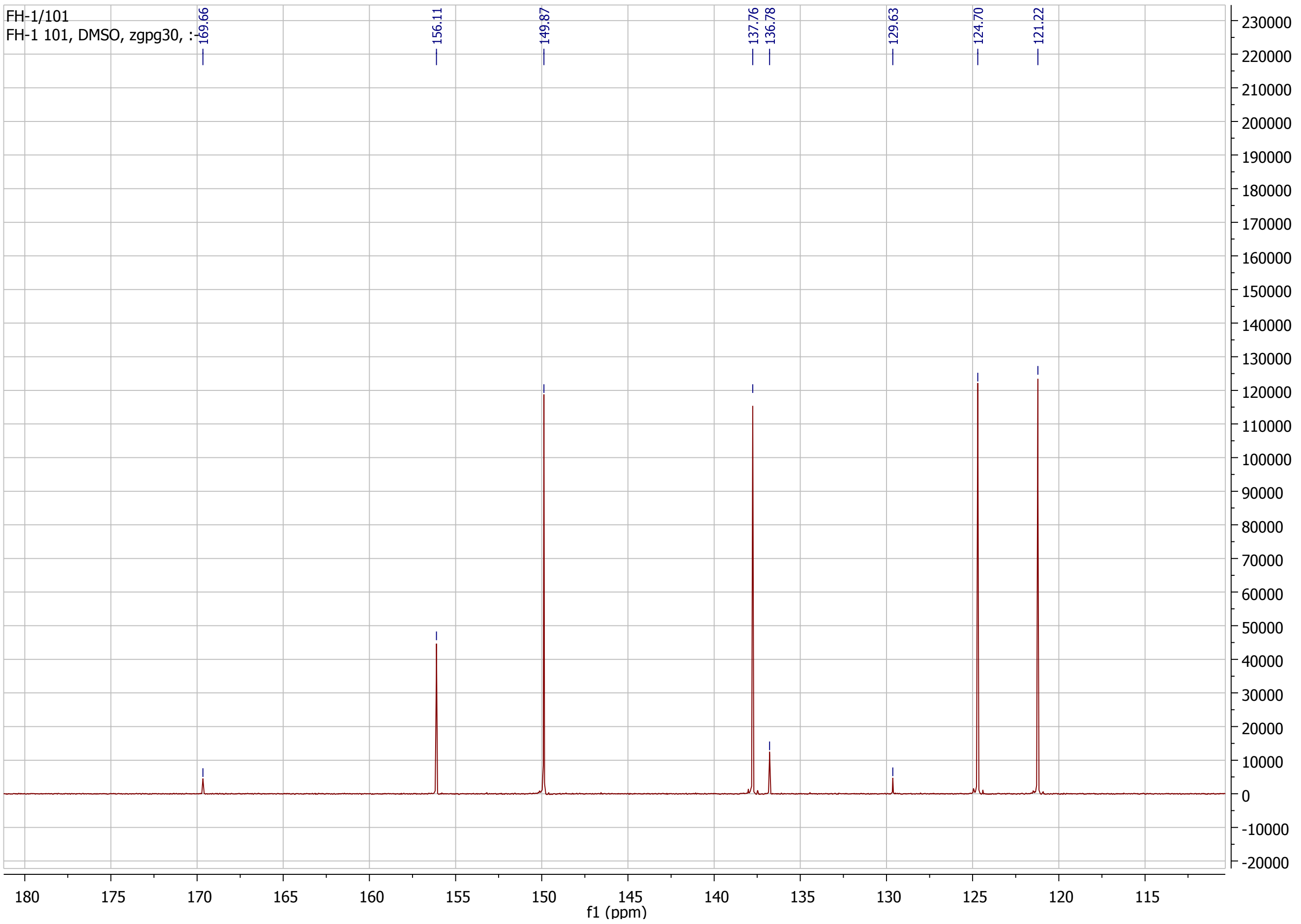
FH-1/101
FH-1 101, DMSO, zgpg30, :-
169.66
156.11
149.87
137.76
136.78
129.63
124.70
121.22
f1 (ppm)

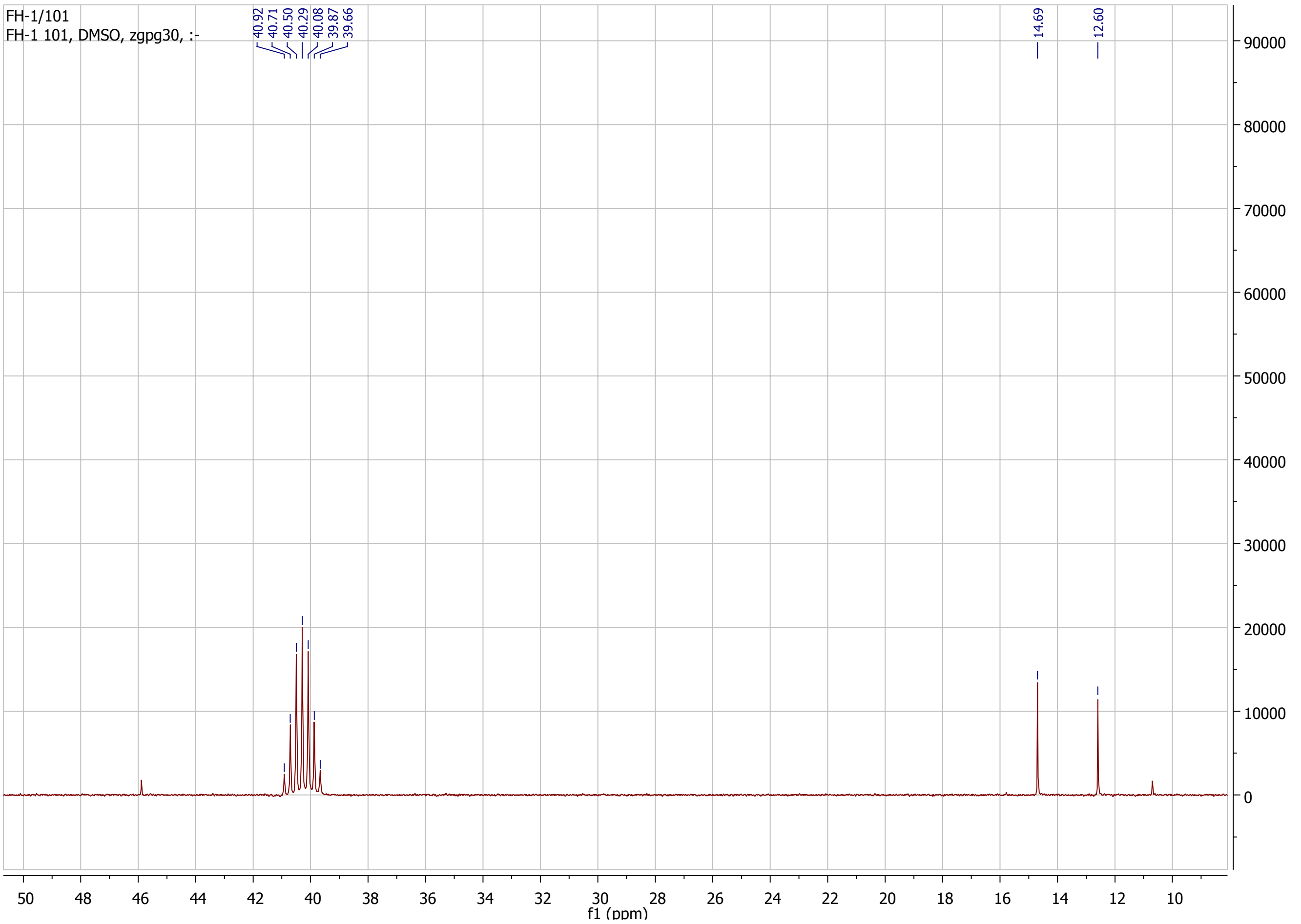
FH-1/101
FH-1 101, DMSO, zgpg30, :-
40.92
40.71
40.50
40.29
40.08
39.87
39.66
14.69
12.60
f1 (ppm)

S3. ^{1}H-NMR spectrum of $[Ru(L_1)_2(NCS)_2]$.

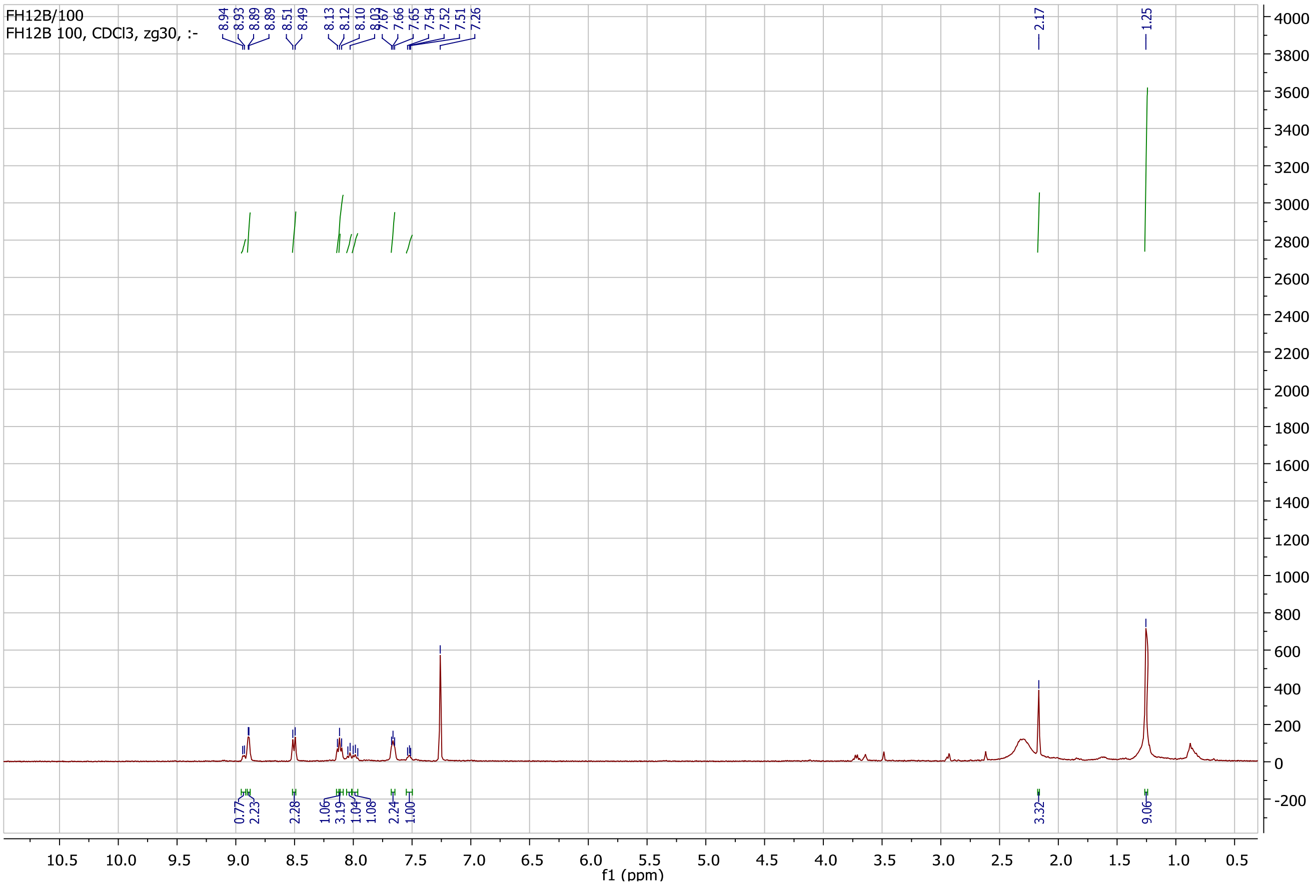

Aromatic region of ^{1}H-NMR spectrum of complex $[Ru(L_1)_2(NCS)_2]$.

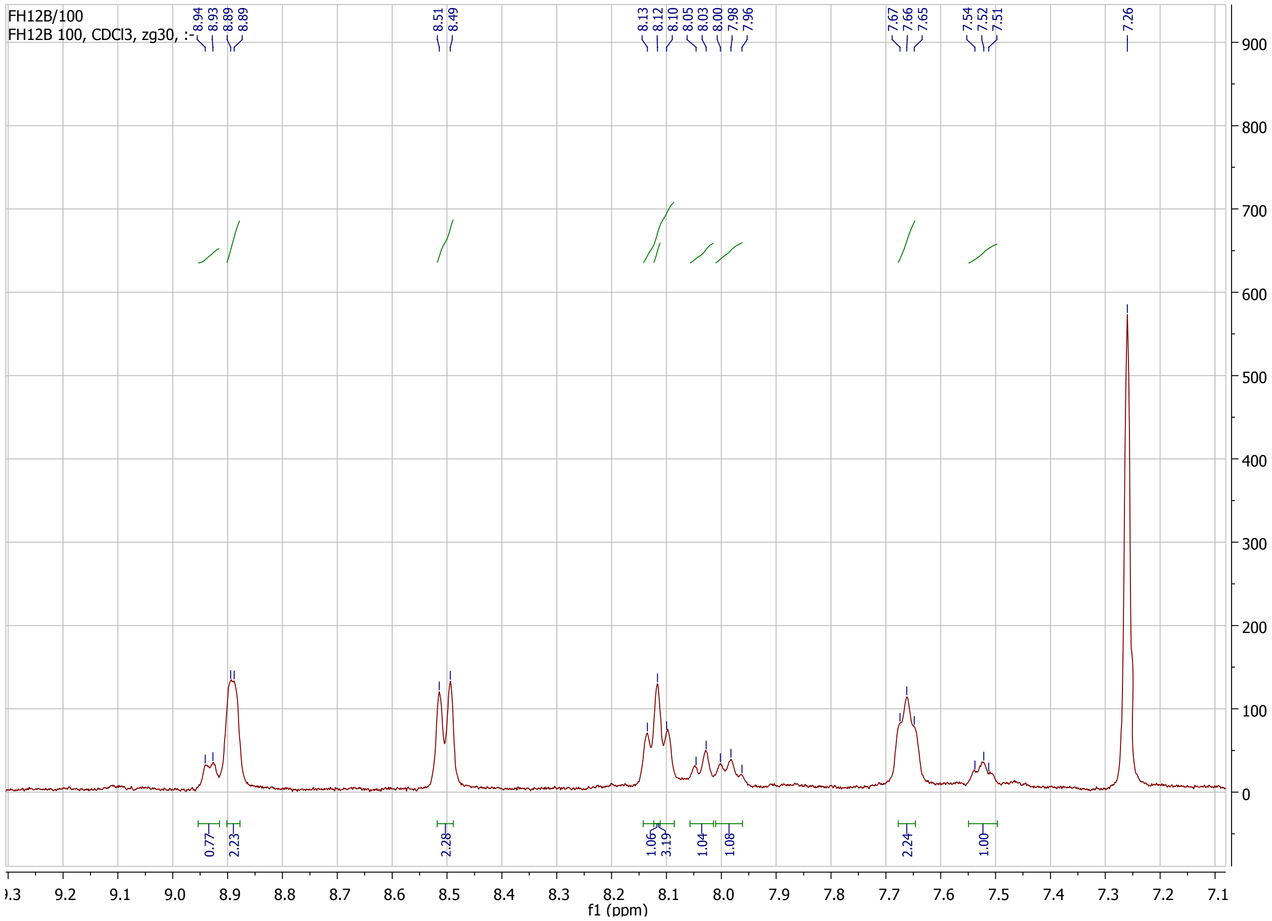

S4. Aromatic region ^{1}H-NMR spectrum of $\mathbf{L_2}$.

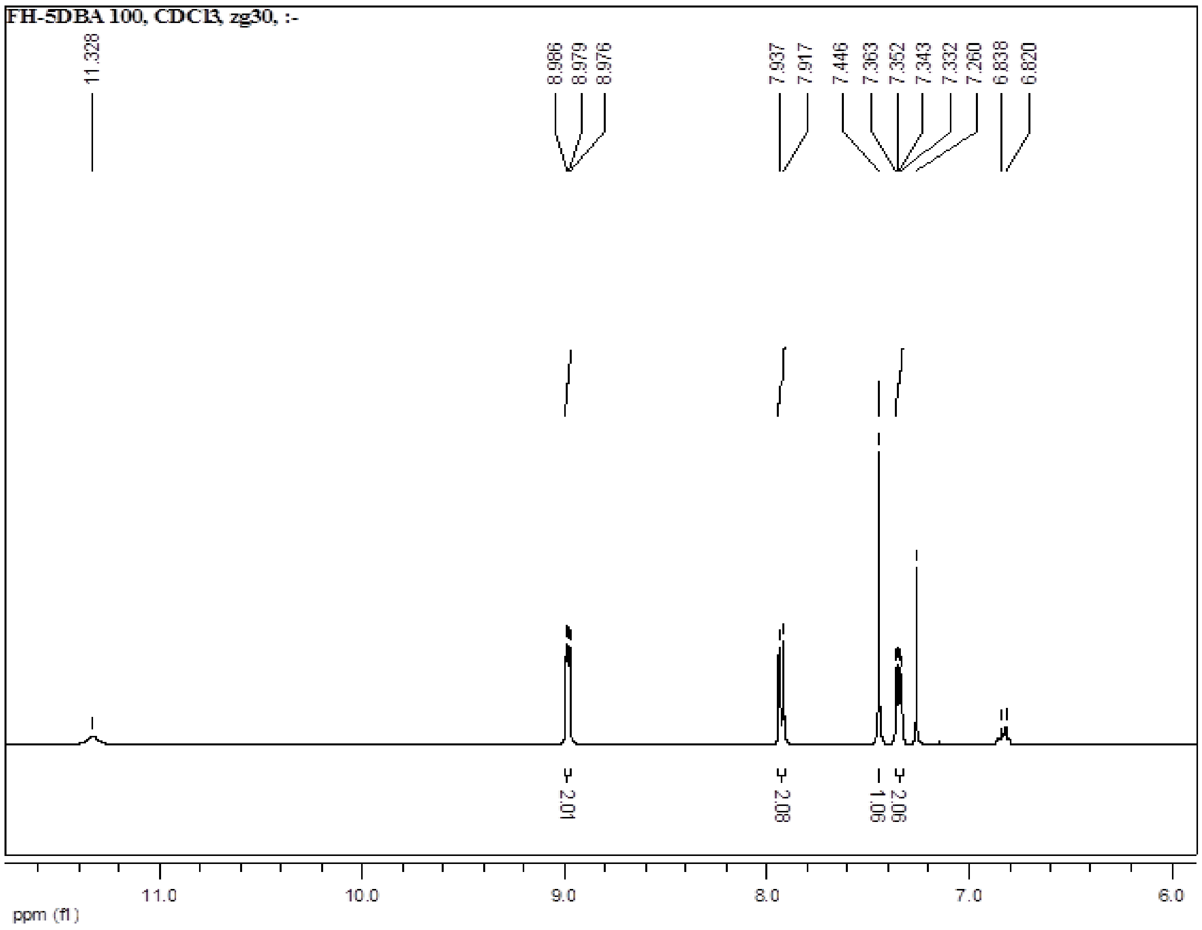

S5. Aliphatic region ^{1}H- NMR of $\mathbf{L_2}$.

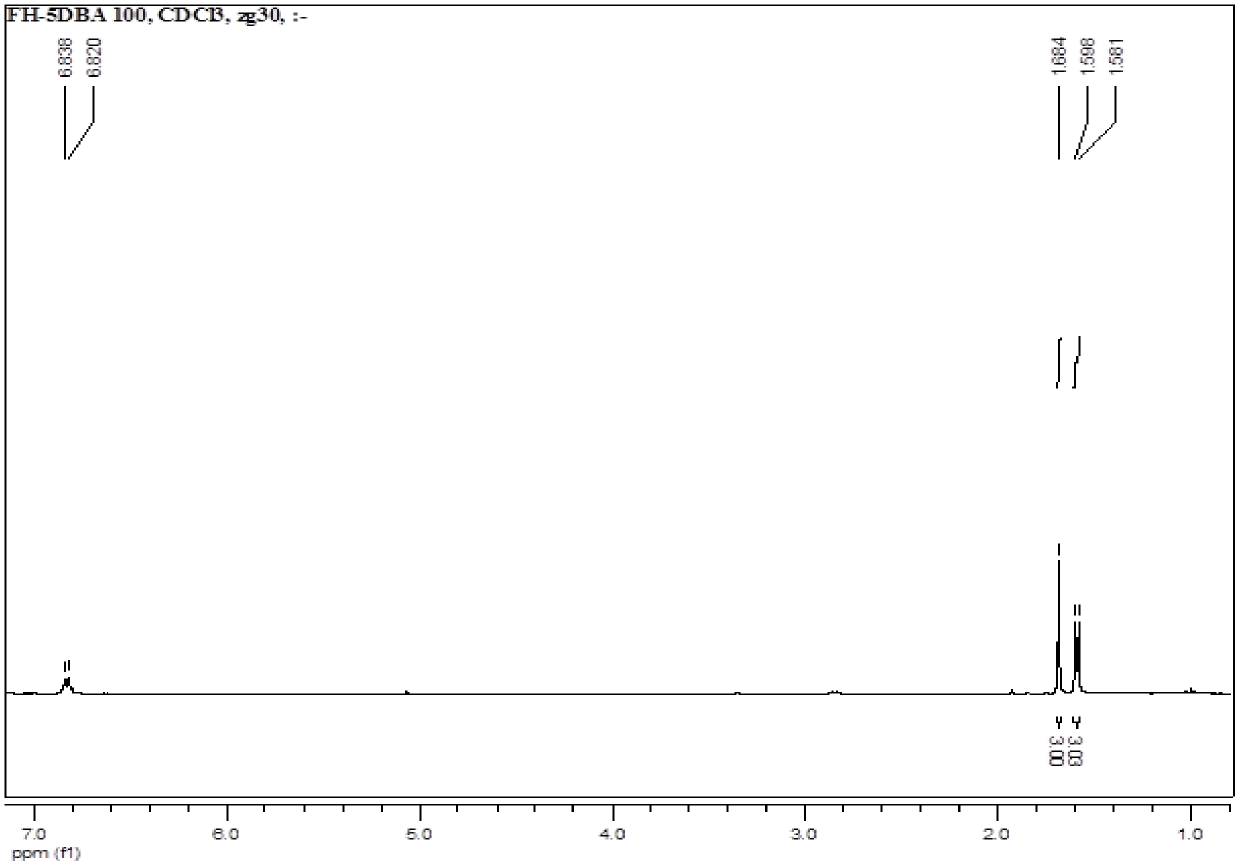

S6. ^{13}C-NMR spectrum of $\mathbf{L_2}$.

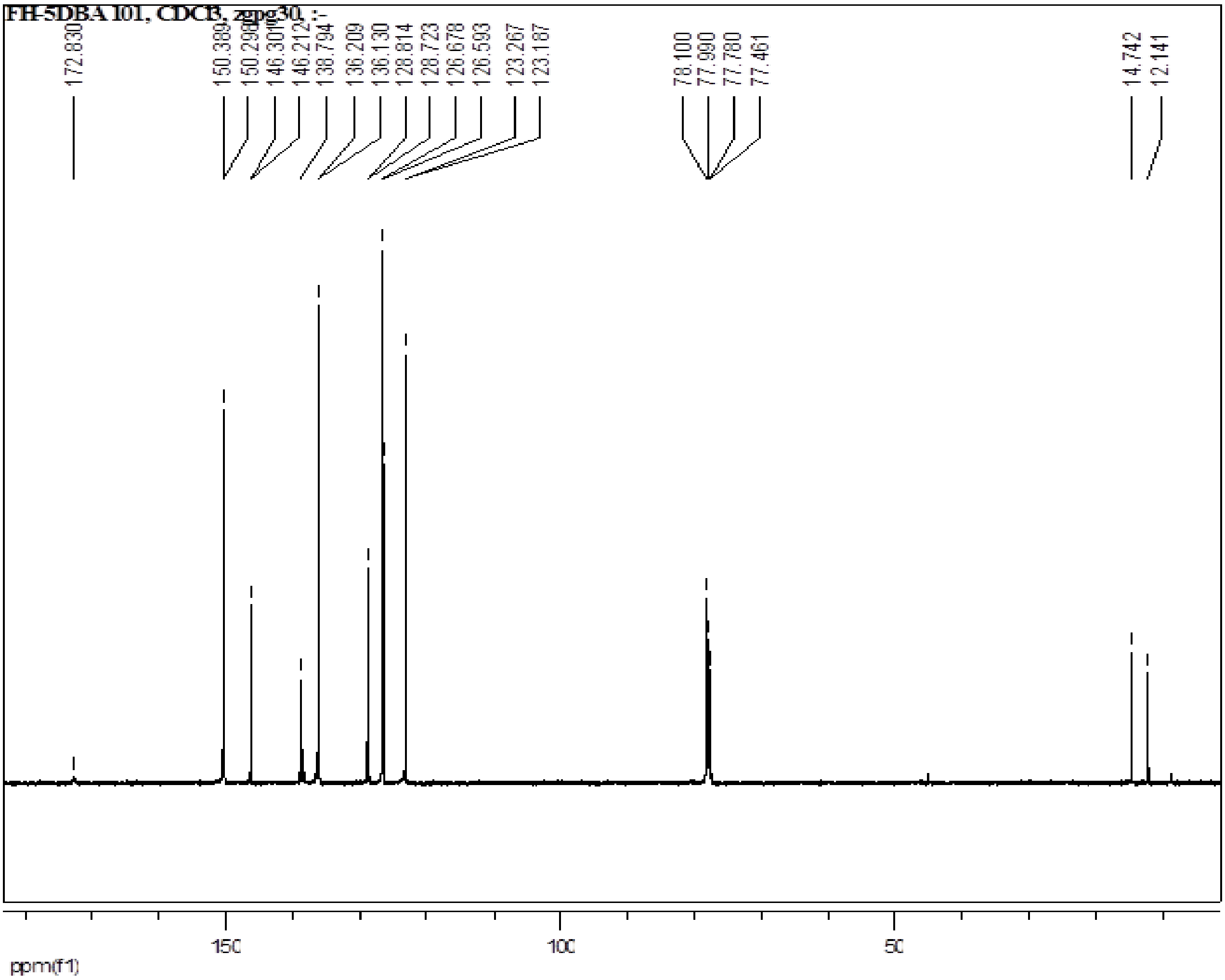

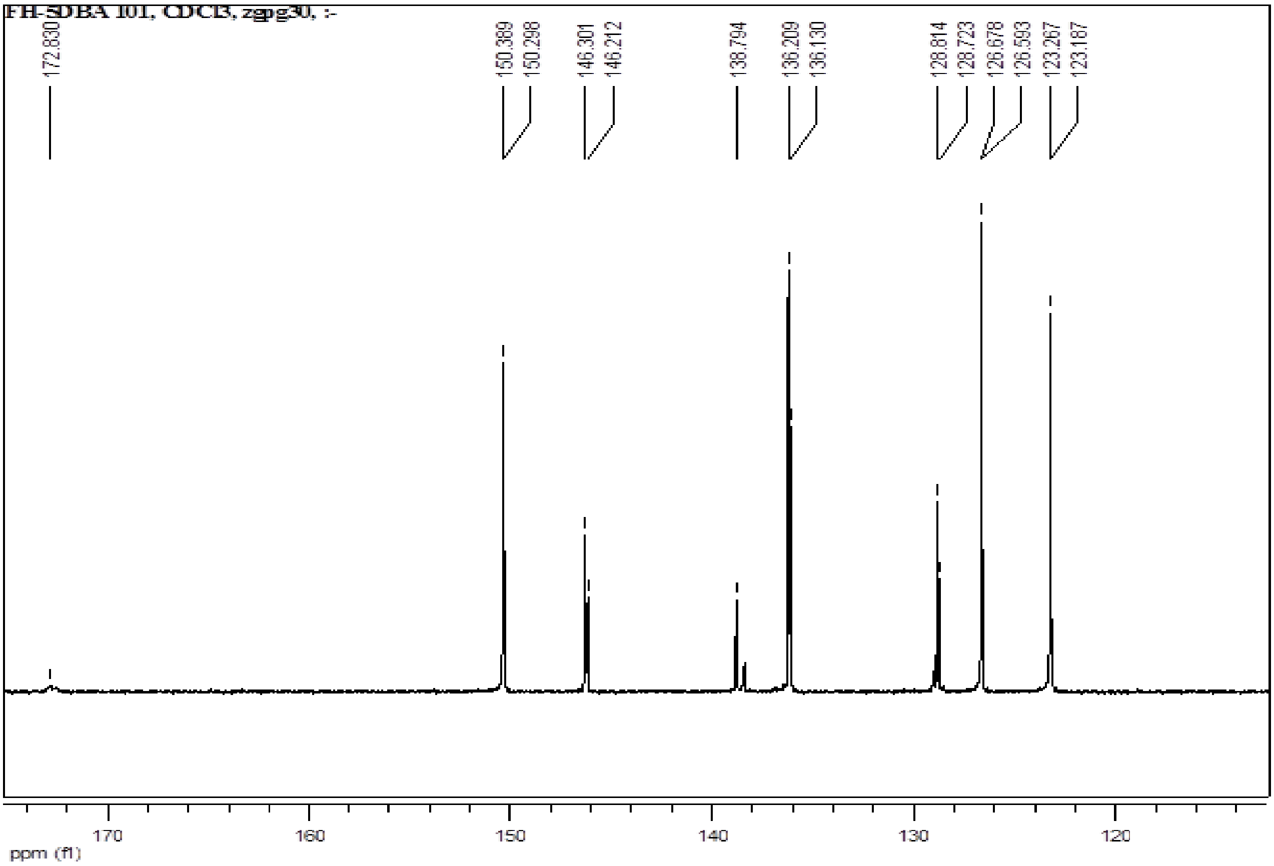

S7. ^{1}H-NMR spectrum of $[Ru(L_2)_2(NCS)_2]$.

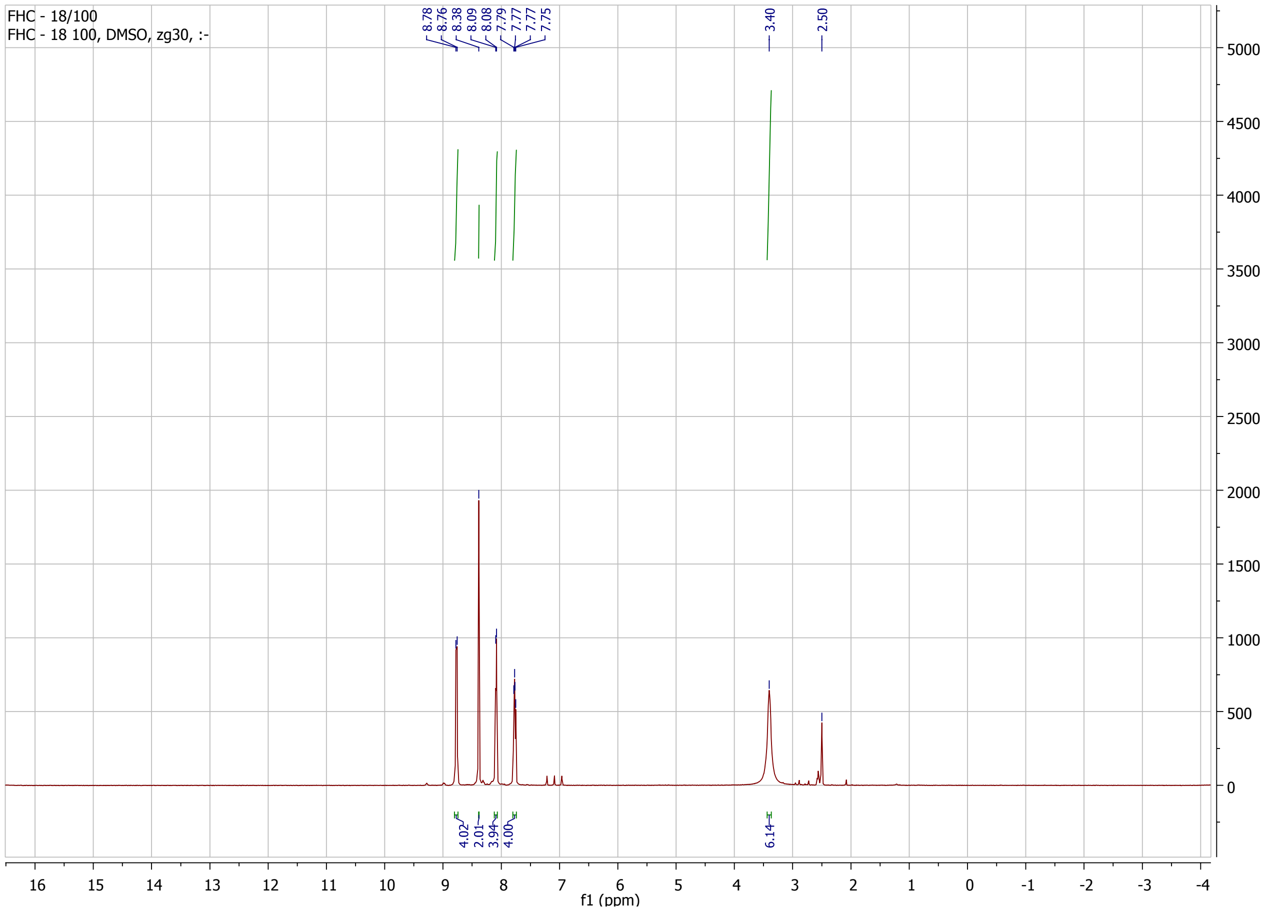

Aromatic region of ^{1}H NMR spectrum of complex $[Ru(L_2)_2(NCS)_2]$.

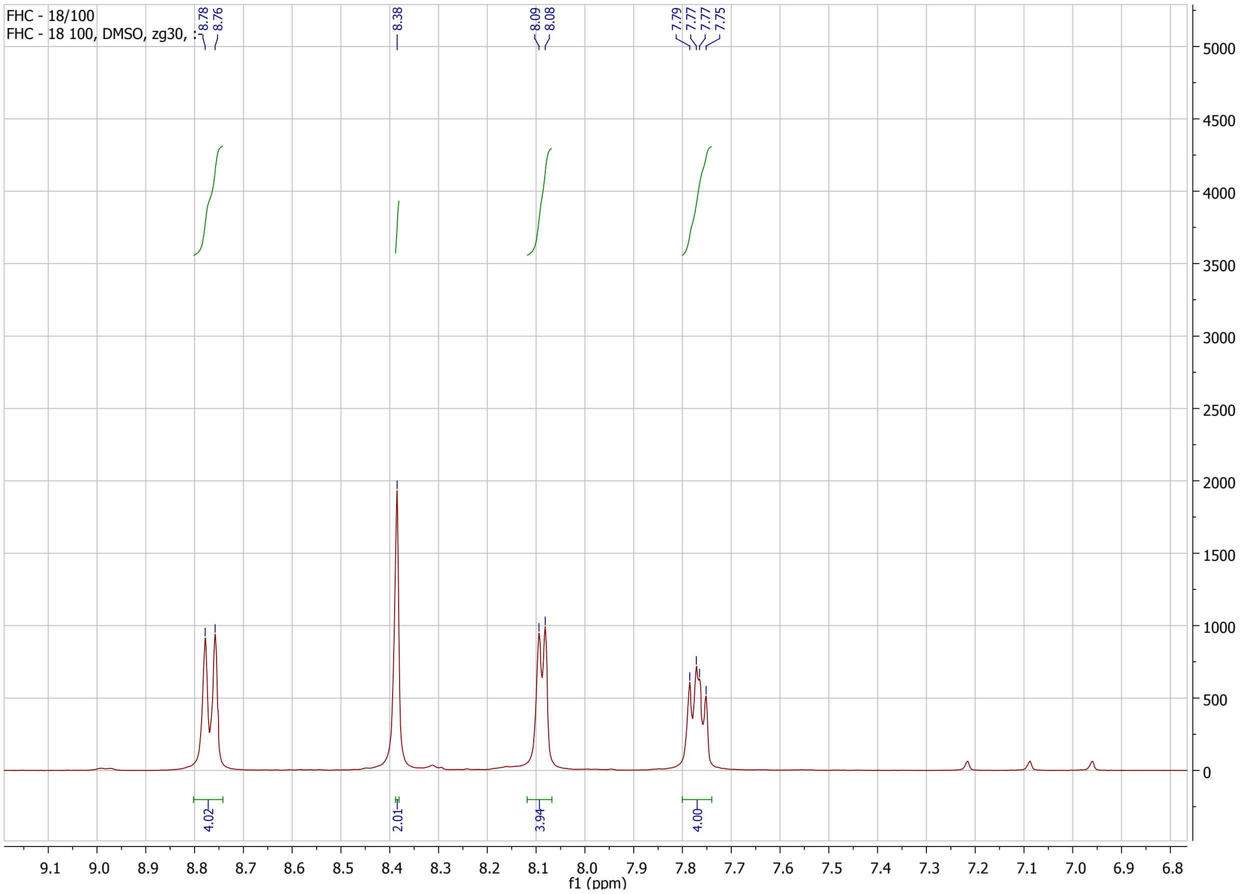

Supplement: Supplementary File 1 [file molecules-16-08353-s001.pdf]
